# Bilateral Wilms Tumor: Looking for the Best Surgical Strategy

**DOI:** 10.1245/s10434-026-19859-9

**Published:** 2026-06-04

**Authors:** Marta Martos Rodríguez, Hélène Sudour-Bonnange, Hubert Ducou Le Pointe, Lauriane Lemelle, Jacinthe Bonneau-Lagacherie, Claudia Pasqualini, Cécile Boulanger, Anne Notz-Carrere, Ludovic Mansuy, Claire Pluchart, Julien Rod, Sarah Jannier, Nicolas Sirvent, Dominique Plantaz, Sébastien Klein, Leslie Andry, Arnauld Verschuur, Benoit Dumont, Georges Audry, Sabine Irtan

**Affiliations:** 1 Pediatric Surgical Oncology Unit, Department of Pediatric Surgery, Vall d’Hebron University Hospital, Barcelona, Spain; 2 Pediatric Surgery Department, Department of Children and AYA Oncology, Centre Oscar Lambret, Lille, France; 3 Pediatric Radiology Department, Sorbonne University, Assistance Publique Hôpitaux de Paris, Armand Trousseau Hospital, Paris, France; 4 SIREDO Oncology Center, Institut Curie, Paris, France; 5 University Hospital of Rennes, Pediatric Oncology and Hematology, Rennes, France; 6 Department of Child and Adolescent Oncology, Gustave Roussy, Villejuif, France; 7 CHU Toulouse, Toulouse, France; 8 CHU Bordeaux, Bordeaux, France; 9 Pediatric Oncology and Hematology Department, University Hospital Center of Nancy, Nancy, France; 10 Department of Paediatric Haematology and Oncology, Centre Hospitalo-Universitaire de Reims, Reims, France; 11 CHU Caen Normandie, Caen, France; 12 Pediatric Oncology Department, University Hospital of Strasbourg, Strasbourg, France; 13 CHU Montpellier, Montpellier, France; 14 Institut Gustave Roussy, GFAOP, Villejuif, France; 15 CHU Jean-Minjoz, Pediatric Oncology and Hematology, Besançon, France; 16 Service d’Oncologie et d’Hématologie Pédiatrique, CHU Amiens, Amiens, France; 17 Pediatric Oncology Department, La Timone, Children’s Hospital, Marseille, France; 18 Pediatric Oncology Department, Leon-Berard Institut, Lyon, France; 19 Neonatal and Visceral Pediatric Surgery Department, Sorbonne University, Assistance Publique Hôpitaux de Paris, Armand Trousseau Hospital, Paris, France

**Keywords:** Wilms tumour, Bilateral disease, Total nephrectomy, Nephron-sparing surgery, Outcome

## Abstract

**Background:**

Management of bilateral Wilms tumor (BWT) to achieve complete tumor control and maintain good long-term renal function is challenging. Nephron-sparing surgery (NSS) allows preservation of renal parenchyma and reduces the risk of renal failure in selected cases. However, there is no standardized surgical strategy. This study analyzed the surgical management for patients included in the SIOP2001 trial and its outcomes.

**Methods:**

This retrospective review analyzed French patients included in the SIOP 2001 protocol for synchronous BWT. Data regarding imaging, surgical strategy, complications, histology, oncologic outcomes, and renal function were collected.

**Results:**

The study included 96 patients (median age, 15 months; range, 0–143 months). They received surgery for 122 Wilms tumors, and 56 (45.9 %) kidneys benefited from NSS. Total nephrectomy (TN) was performed in 27/35 (77.1 %) and 33/76 (43.4 %) of the histologies non-sensitive to chemotherapy (stromal and diffuse anaplasia, respectively). For bilateral surgery, the study found a trend to more TNs, tumoral rupture, and death among the patients who had surgery in a single stage (*p* > 0.05). Tumor rupture was associated with higher tumoral volume (*p* < 0.05). In a review of preoperative images, vascular contact was the main factor associated with TN. Additional chemotherapy did not seem to significantly reduce this contact or the number of TNs in these patients.

**Conclusion:**

Whenever possible, NSS should be prioritized for patients with BWT. In this study, the most limiting factor was contact with vascular structures. Additional chemotherapy for tumors with non-sensitive histology or extensive vascular contact had little impact on the final surgical strategy. Staged surgery should be considered as it appeared to reduce surgical morbidity.

Nephroblastoma or Wilms tumor (WT) is the most common renal tumor in children and accounts for the fourth malignancy in pediatric patients. Sporadic forms account for 90 % of cases. Cases of genetic predisposition are mostly constitutionals, familial forms being very rare (<1 %).^1^ Bilateral disease is found in 5 %–8 % of patients, most often (2/3) being a synchronic form. Patients presenting with bilateral WT (BWT) are younger, with a median age of 24 months at diagnosis (vs 38 months for unilateral forms). There is a higher incidence of genetic predisposition including Beckwith-Wiedemann (BWS), Denis-Drash (DDS), Frasier, and WAGR syndromes.

Nearly all cases of BWT are associated with the presence of residual embryonic cells known as nephrogenic rests (NRs), which are considered precursors of WT.^2^ Thus, the presence of NR in the contralateral kidney in a case of unilateral WT increased the risk of metachronous disease.^2,3^

Prognosis in BWT has improved due to optimization of the chemotherapy protocols and the surgical techniques. In recent protocols, long-term overall survival is about 80 %,^4^ approaching rates of unilateral cases. However, due to the higher intensity of medical and surgical strategies, morbidity associated with the treatment remains important. This is especially true for end-stage renal disease, not only due to loss of renal tissue, but probably also due to an intrinsic abnormal development of mature renal parenchyma.^5^

Therefore, the purpose in treating patients with bilateral disease is surely to achieve control of the oncologic disease, but also to prevent renal failure. With this aim, recent protocols include strategies based on prolonged chemotherapy regimens with the goal of reducing tumor volume before surgery. Regarding surgical management, nephron-sparing surgery (NSS) by partial nephrectomy, wedge resection, or enucleation has demonstrated favorable oncologic and functional outcomes. The perfect time to perform the surgery as well as the surgical strategy remains a major clinical challenge. Due to the complexity and the rarity of these cases, it is of utmost importance for these patients to be managed by experienced teams after multidisciplinary discussion of each particular case.

Despite the significant improvements in treatment achieved in recent years, no standardized surgical strategy currently exists for the management of patients with BWT. The purpose of this study was to analyze the surgical strategy followed for patients treated in the framework of the SIOP 2001 trial with the aim of proposing a structured approach to surgical decision-making for children with BWT within the chemotherapy protocol, particularly regarding timing of surgery and nephron preservation.

## Materials and Methods

The SIOP WT 2001 study, an international multicentric study performed between 2001 and 2015, enrolled children with a diagnosis of renal tumors. The current study was a retrospective review of patients treated in France for synchronous bilateral disease (stage V) who were included in the SIOP 2001 protocol. It should be noted that a subset of patients from this national cohort has previously been reported in a publication.^4^; However, the current study addresses a different research question, focusing specifically on surgical timing and strategy. We included patients registered as having bilateral disease, and we considered either BWT, unilateral WT with contralateral NR, or bilateral hyperplastic NR. Metachronous disease was excluded from the analysis as well as renal tumors other than WT.

According to the SIOP 2001 WT guidelines, the patients with stage V disease received preoperative vincristine and actinomycin D (VA) as long as the tumor showed signs of regression with imaging reassessment every 4 weeks. The goal was the bilateral NSS as much as possible. If response to VA cycles was insufficient and/or NSS still was not achievable, VA treatment was intensified with doxorubicin (VAD) or other chemotherapies. Based on information collected in the SIOP 2001 registration form, clinical data regarding age at diagnosis, clinical presentation, congenital anomalies, genetic disorders, and tumoral volume were recorded.

Patients were classified according to the type of surgery they received: bilateral total nephrectomy (TN), unilateral TN and contralateral NSS, bilateral NSS, unilateral TN, or unilateral NSS. Information about the chemotherapy regimen used, timing of surgery, post-surgical stage, and histologic records was analyzed. Outcomes in terms of survival, recurrence, and renal function were studied according to the type of treatment received.

For a subset of patients, we centrally reviewed the preoperative images to analyze more specifically the characteristics of the nodules (size, vascular involvement, contact with the urinary tract, and presence of lymph nodes). The results from this part of the cohort are highlighted in a specific section.

Statistical analysis was performed using IBM SPSS Statistics 26.0 software. Variables are expressed as mean ± standard deviation for continuous variables or as percentage for categorical variables. The Student’s *t* test was used for the comparison of continuous variables. Pearson’s chi-square was used for categorical variables. Analysis of variance (ANOVA) was used to compare variances across various groups. A *p* value lower than 0.05 was considered statistically significant.

## Results

The SIOP WT 2001 trial in France retrospectively registered 96 patients for synchronous bilateral disease: 34 with bilateral WT, 39 who had unilateral WT with contralateral NR, and 19 with bilateral hyperplastic NR. For the remaining four patients, the distribution was not specified, and they were treated in 23 different oncologic centers. The median age at presentation was 23 months (range from antenatal to age of 11.9 years). The sex ratio was 1.13 (51 boys and 45 girls). Of the 96 patients, 23 (23.9 %) had an associated genetic predisposition syndrome, congenital anomalies, or both; 2 had WAGR syndrome; 10 had BWS; 6 had urogenital anomalies; 4 had hemi-hypertrophy; and 1 had renal dysplasia. Eight patients presented with metastatic disease (1 pulmonary and 1 hepatic and pulmonary). Mean tumoral volume measured on the initial computed tomography (CT) scan was 288.26 ml (342.82 ml for the right tumors and 232.47 ml for the left tumors).

Of the 96 patients, 8 (8.3 %) underwent a bilateral TN, 30 (31.2 %) had unilateral TN and contralateral NSS, 6 (6.2 %) had bilateral NSS, 14 (14.6 %) had unilateral NSS, and 20 (20.8 %) had unilateral TN. In three cases, the surgical procedure was not specified. The remaining 15 patients (15.6 %) did not receive surgery. All the groups had similar demographic characteristics (Table 1). Of 122 surgically managed kidneys, 56 (45.9 %) received NSS (8 enucleations, 13 wedge resections, 35 partial nephrectomies).

**Table 1:** Demographic characteristics by surgical intervention group

	Bilateral nephrectomy (*n* = 8) *n* (%)	Bilateral NSS (*n* = 6) *n* (%)	Unilateral nephrectomy + contralateral NSS (*n* = 30) *n* (%)	Unilateral nephrectomy (*n* = 20) *n* (%)	Unilateral NSS (*n* = 14) *n* (%)	Non operated (*n* = 15) *n* (%)
Median age: months (range)	20.6 (2–68)	18 (5–46)	23.5 (5–60)	29.5 (7–143)	24.5 (prenatal: 65)	16.5 (prenatal: 49)
Sex ratio (boys:girls)	1:1	1:1	1,3:1	3:1	1:1.3	1:1.8
Genetic predisposition syndrome and/or congenital anomalies	3 (37.5)	2 (33.3)	2 (6.6)	4 (20)	4 (28.6)	6 (40)
Bilateral nephroblastoma	6 (75)	5 (83.3)	23 (76.6)	0	0	0
Unilateral nephroblastoma + contralateral NR	1 (12.5)	1 (16.7)	6 (20)	20 (100 +)	11 (78.6)	0
Bilateral NR	1 (12.5)	0	0	0	3 (21.4)	15 (100)
Metastatic disease	0	0	4 (13.3)	3 (15)	1 (7.1)	0
Mean right tumor volume (ml)	393.6 ± 275.4	133.95 ± 172.6	287.98 ± 488.2^a^	611.5 ± 286.4	156.92 ± 136.3	218.2 ± 213.1
Mean left tumor volume (ml)	315.50 ± 230.4	92.25 ± 128.2	312.61 ± 303.1^a^	474.76 ± 292.5	203.75 ± 264.5	47.4 ± 88.7

NR, nephrogenic rest; NSS, nephron-sparing surgery

^a^401.8 ± 342.2 ml for the total nephrectomy group and 172.8 ± 220.4 ml for the NSS group

The mean duration of preoperative chemotherapy was 96 days (range, 14–468 days). All the patients received vincristine and actinomycin D. Doxorubicin was administered to 20 (20.8 %) patients, and 22 (22.9 %) patients also received other drugs. In those patients who received 4 to 8 weeks of presurgical chemotherapy, we found TN in 51.5 % (35/68 kidneys) compared with 42.8 % (27/63 kidneys) in those who received 12 or more weeks of presurgical chemotherapy (*p* > 0.05). The percentage of NSS did not evolve during the time period of SIOP 2001.

Among the 44 patients who received surgery for both kidneys, 30 (68.2 %) had one-step bilateral surgery, and 14 (31.8 %) had two-step surgery (mean time of 93 days between the two surgeries). For those who had surgery in two steps, the first procedure was a TN in 11 cases and an NSS in 3 cases, whereas the second procedure was a TN in 4 cases (2 patients having had a TN in the first step) and a NSS in 10 cases. Although the differences in the outcomes were not significant, twice as many tumoral ruptures and more deaths occurred in the one-step group (Table 2). Overall, 8 bilateral total nephrectomies were performed: 2 (14.3 %) of 14 performed in two steps and 6 (20 %) of 30 performed in one step (*p* > 0.05).

**Table 2:** Outcomes for one- vs two-step surgery (bilateral surgery)

	One step (*n* = 30) *n* (%)	Two steps (*n* = 14) *n* (%)	*p* Value
Tumoral rupture	4 (13.3)	1 (7.1)	NS
Local stage III	7 (23.3)	4 (28.6)	NS
Normal renal function	27 (90)	12 (85.7)	NS
Abnormal renal function	3 (10)	2 (14.3)	NS
Renal transplantation	5 (16.7)	3 (21.4)	NS
Disease relapse	3 (10)	2 (14.3)	NS
Free of disease	27 (90)	14 (100)	NS
Alive with disease	0	0	NS
Dead	3 (10)	0	NS

NS, not significant

We found (12/60) high-risk histology in 20 % of the kidneys receiving TN versus 11,7 % (6/51) of those receiving NSS. Intermediate risk was found for 33.3 % (20/60) of the stromal type among the kidneys receiving TN versus 13.7 % (7/51) for those receiving NSS. Analysis of the surgical procedure among histologic subtypes non-responsive to chemotherapy found that 71,4 % (20/28) of the stromal subtype and 100 % (7/7) of the diffuse anaplasia subtype received a TN (Table 3).

**Table 3:** Histology by type of surgery

	Total nephrectomy (*n* = 60)	NSS (*n* = 51)	*p* Value
NR	2	10	
Low risk	2	6	
Intermediate risk * Stromal * Other subtypes	44 20 24	29 8 21	NS
High risk * Blastemal * Diffuse anaplasia	12 5 7	6 6 0	0.025

NSS, nephron-sparing surgery; NR, nephrogenic rest

Nine patients had intraoperative tumor rupture: six during a TN and three during NSS (2 partial nephrectomies and 1 wedge resection). The mean volume of the ruptured tumors was 545.71 ml, which was higher than the tumoral volume of non-ruptured tumors (*p* < 0,05). A total of 22 patients were classified as local stage III after surgery (Table 4).<T4>

**Table 4:** Surgical results and outcomes by interventional group

		Bilateral nephrectomy (*n* = 8)	Bilateral NSS (*n* = 6)	Unilateral nephrectomy + contralateral NSS (*n* = 30)	Unilateral nephrectomy (*n* = 20)	Unilateral NSS (*n* = 14)	*p* Value
Tumoral rupture		1	0	3	2	3	NS
Local stage III		2	1	9	6	4	NS
Final histology	NR	1	0	0	0	3	0.03
	Low risk	0	0	2	0	2	NS
	Intermediate risk	5	4	21	15	6	NS
	High risk	2	2	5	5	2	NS
Disease relapse		0	1	4	7	2	NS
Free of disease		8	5	27	17	11	NS
Alive with disease		0	0	0	1	2	NS
Dead		0	1	2	2	1	NS
Normal renal function		0	6	23	17	14	<0.05 (for bilateral nephrectomy and unilateral NSS)
Abnormal renal function		0	0	4	1	0	NS
Renal transplantation		8	0	2	2	0	<0.05 (for bilateral nephrectomy)

NSS, nephron-sparing surgery; NS, not significant; NR, nephrogenic rest

Postoperative chemotherapy was performed for 60 patients (26 VAs, 13 VADs, and 21 other regimens). Postoperative radiotherapy was performed in 20 cases, 17 for stage III local disease, 1 for stage II disease with high-risk histology, 1 for reception of biopsy at diagnosis, and 1 for local relapse.

The median follow-up period was 82.16 months. Survival, disease relapse, and renal function outcomes are summarized in Table 4. All deaths were due to tumor progression. Of the surgically managed patients, 14 (17.9 %) relapsed (9 local and 5 metastatic relapses). The mean time from diagnosis to relapse was 38 months. All the patients who received bilateral nephrectomy had a renal transplantation. Renal function was significantly better for the patients who received unilateral NSS on only one side.

Of the 23 patients who presented with a genetic predisposition syndrome, 8 relapsed (*p* = 0.03). No differences in terms of renal function or mortality were found between the patients with and those without genetic predisposition syndrome. It should be noted, however, that 4 of the 12 patients who received a kidney transplantation were classified as having congenital urogenital malformation. No differences were found in the number of NSSs performed or the risk of recurrence in these patients.

### Analysis of Patients wth Reviewed Images

We were able to review the preoperative images of 49 (51 %) of the 96 patients in our study. In the remaining cases, we did not have access to the images due to difficulties because of transfer from other centers or because they were before the implementation of the Picture Archiving and Communication System (PACS).

Of the 49 patients, 20 had unilateral surgery, 22 had bilateral surgery (16 in one step and 6 in two steps), and 4 did not receive surgery. The procedure for the remaining three patients was not specified.

Surgical procedures included 39 TNs and 25 NSSs. The preoperative radiologic parameters that mainly determined the surgical decision were the contact of the tumor with the urinary system and the renal pedicle. Kidneys that received a TN presented nodules having more contact with the vascular pedicle associated with a contact of the tumor with the urinary system (*p* < 0.001), whereas NSS was more often performed when the tumor was in contact only with the urinary system (Table 5).

**Table 5:** Radiologic characteristics for total nephrectomy vs NSS (for patients with reviewed images)

	Total nephrectomy (*n* = 39)	NSS (*n* = 25)	*p* Value
Mean tumor volume (ml)	301.2 ± 62.8	75.2 ± 35.6	0.02
Median no. of nodes (range)	1 (1–7)	1 (1–4)	NS
Contact with the urinary system	28	14	NS
Vascular involvement	30	4	<0.001
Contact with the urinary system only	2	10	<0.001
Vascular involvement only	4	0	NS
Contact urinary system and vascular involvement	26	4	<0.001
No contact with the urinary system nor vascular involvement	7	11	0.02
Venous thrombosis	5	0	NS

NSS, nephron-sparing surgery; NS, not significant

For 32 patients, we also were able to review the image at diagnosis, which allowed us to evaluate the tumor response to preoperative treatment. Among 14 tumors with vascular contact (10 TNs and 4 NSSs), 7 had surgery at 4 to 8 weeks. The remaining seven patients received additional treatment up to 12 weeks, reaching release of vascular involvement in two of them (1 TN and 1 NSS) (*p* > 0.05).

We found no differences in size or proximity with the urinary and vascular structures between those who had surgery in one step and those with surgery in two steps (Table 6). Six patients had surgery in two steps, with a mean time between surgeries of 74 days (range, 31–123 days). Five of these patients had one or two small nodules in the kidney treated during the second stage of surgery that did not disappear after prolonged administration of chemotherapy. The remaining patient had a large tumor volume on both sides. The volume of the tumor on the left side did not decrease with chemotherapy, so a TN was performed. The right tumor presented with a good response and received surgery after 65 days of additional chemotherapy so he could benefit from NSS. In all these patients, histology confirmed the presence of WT. The side that had surgery first was always the largest and/or the one with the greatest involvement of the vascular pedicle (patients with venous thrombosis always had surgery first). All those with venous thrombosis had surgery in two steps (Table 7).

**Table 6:** Radiologic characteristics for those who had surgery in one step vs two steps (for patients with reviewed images)

	One step (*n* = 16)	Two steps (*n* = 6)	*p* Value
Mean tumor volume (ml)	170 ± 83.4	299 ± 107.4	NS
Median no. of right nodes (range)	1 (1–2)	1 (1–2)	NS
Median no. of left nodes (range)	2 (1–3)	1 (1–2)	NS
Contact with the urinary system	11 (17/32 kidneys)	5 (9/12 kidneys)	NS
Vascular involvement	13 (17/32 kidneys)	4 (5/12 kidneys)	NS
Venous thrombosis	0	3	0.01

NS, not significant

**Table 7:** Characteristics for kidneys treated with surgery on the first vs the second step for the six patients who had surgery in two steps (for patients with reviewed images)

		First step (*n* = 6)	Second step (*n* = 6)	*p* Value
Mean tumor volume (ml)		605.26 ± 238.6	91.98 ± 52.4	0.04
Median no. of nodes (range)		1 (1–2)	1 (1–2)	NS
Contact with the urinary system only		0	5	<0.01
Vascular involvement only		1	0	NS
Contact with the urinary system and vascular involvement		4	1	NS
No contact with the urinary system nor vascular involvement		2	0	NS
Venous thrombosis		3	0	NS
Total nephrectomy		6	0	0.002
Final histology	NR	0	0	NS
	Low-risk	1	1	NS
	Intermediate-risk	3	4	NS
	High-risk	1	0	NS

NS, not significant; NR, nephrogenic rest

## Discussion

Survival of patients with bilateral Wilms tumor has improved significantly during the last decades due to advances in chemotherapy protocols and surgical management.^6^ Nevertheless, treatment-related morbidity remains high, particularly among patients with the risk of end-stage renal disease, which is the second leading cause of death for survivors of BWT, reaching rates of 15 % in 20 years (vs 1.3 % in the unilateral disease group).^7,8^

Current SIOP and Children’s Oncology Group (COG) recommendations encourage the use of NSS whenever feasible.^9–11^ In our series, 73 % of the patients treated with surgery had NSS at least on one side, and 6.2 % had bilateral NSS. One of the great challenges is selecting the appropriate cases to receive NSS without increasing the risk of surgical complications or relapse. No surgical guidelines exist that offer clear indications, leaving the decision to the surgical team.

Several authors have published good results with the use of NSS for WT.^12,13^ Davidoff et al.^14^ reported 92.9 % of bilateral NSS, with overall survival of 85.7 % and no patient experiencing end-stage renal disease. Our series of bilateral disease had an NSS rate of 45.9 %. The tumor volume was the only factor showing significant association with a higher risk of tumor rupture.

In our study, with a trend toward a higher rate of tumor rupture, TN and mortality were observed in the one-step group. However, these differences were not statistically significant, and all deaths were related to tumor progression rather than surgical complications. Tumor characteristics were comparable between the groups except for venous thrombosis, which was<AQ2> always treated with surgery in two steps. Murphy and Davidoff^15^ support a staged approach for tumors with intravascular thrombosis to preserve renal function before contralateral manipulation.

The question arises about which kidney should receive surgery first and which could benefit from longer chemotherapy treatment with the aim of increasing the number of NSSs. In the current series, the kidney that received surgery first was significantly larger and had greater vascular involvement, which is consistent with the finding of other authors.^16^ The side with venous thrombosis always received surgery first. Kidneys treated in the second stage mostly had small nodules that persisted after chemotherapy and were confirmed as WT on final histology. This highlights the importance of being alert to small nodules that, although suggestive of NR, persist after neoadjuvant therapy. We did not find a greater prevalence of high-risk histology in second-stage tumors.

Contact with vascular structures appeared to be a major determinant of TN in our cohort, consistent with previous literature.^17^ Prolonged neoadjuvant treatment did not clearly reduce this contact, and the proportion of total nephrectomies remained high. All patients with venous thrombosis received a total nephrectomy. Although NSS has been reported for selected hilar tumors in expert centers, this strategy may not be broadly generalizable.^18^

Regarding chemotherapy duration, we did not identify differences in TN rates between patients receiving 4–8 versus 12 weeks of neoadjuvant treatment. However, stromal subtype and diffuse anaplasia were significantly more frequent among TN cases, consistent with their lower chemosensitivity. These findings may suggest that prolonging chemotherapy for clearly non-responsive tumors does not substantially modify surgical management.

Patients who present with genetic alterations that predispose to the development of BWT deserve special mention. For them, it is particularly important to try to preserve renal parenchyma due to the risk for the development of metachronous tumors in addition to the higher prevalence of renal failure inherent to their condition.^7,19^

Our study had limitations such as its retrospective nature, the difficulty accessing information in some cases, and the small number of available preoperative images. Furthermore, we were unable to obtain information about the details of the surgical intervention.

Overall, our findings support individualized surgical planning based on tumor anatomy, vascular involvement, and multidisciplinary team assessment. Staged surgery may facilitate NSS in complex cases, but the decision should be guided by tumor characteristics and response to chemotherapy rather than assumed outcome differences between one- and two-step approaches.

Further strategies, both surgical and systemic, are needed to increase the feasibility of NSS for patients with BWT.

Based on our findings, the following considerations may be proposed:

- Patients with BWT represent a complex group of patients who must be treated in reference centers so that they can receive the best treatment based on the decisions made by a multidisciplinary committee.
- NSS should be considered, When technically possible, NSS should be considered for tumors located peripherally and at a distance from the renal hilum.
- For tumors that respond to neoadjuvant treatment, chemotherapy can be continued for up to 12 weeks to reduce renal parenchymal resection.
- In tumors with non-sensitive histology (stromal and diffuse anaplasia) and/or extensive vascular contact at diagnosis, prolonging chemotherapy may not substantially modify the final surgical approach.
- Staged surgery should be considered to reduce surgical aggression, especially in the case of more complex surgeries such as those involving the presence of venous thrombosis.
- Persistent small nodules after chemotherapy warrant careful evaluation and should not be systematically considered nephrogenic rests.
- Further strategies, both surgical and systemic, are needed to try to increase NSS in patients with BWT.

**Fig. 1:**
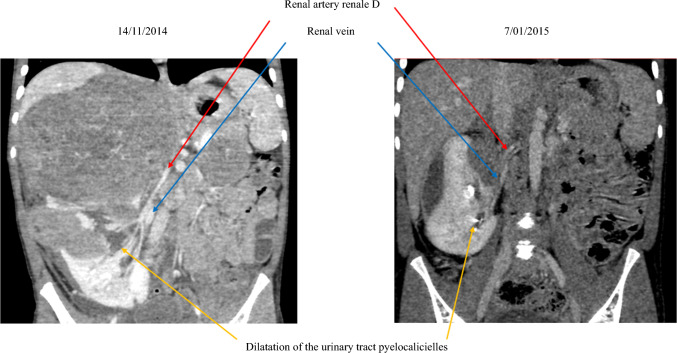
Right Wilms tumor with vascular involvement and dilatation of the urinary tract that persists after six weeks of chemotherapy

**Fig. 2:**
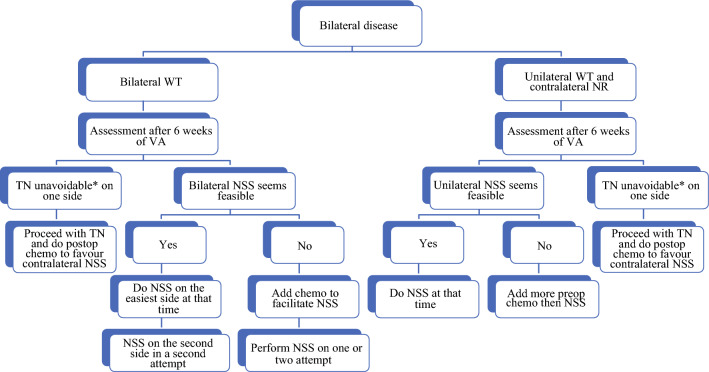
Proposed algorithm for the management of bilateral Wilms tumors. TN=total nephrectomy. NSS: Nephron sparing surgery.* We consider TN unavoidable when there is vascular involvement

Based on these considerations, we propose the treatment algorithm for BWT shown in Fig. 1 In all cases, a careful response evaluation is performed after the corresponding neoadjuvant chemotherapy. If TN is considered unavoidable (extensive vascular contact), it may be performed at that stage followed by chemotherapy with the aim to attempt an NSS on the contralateral side. On the other hand, if bilateral NSS seems possible after evaluation, NSS on one side can be considered at this time and on the other side after prolonged chemotherapy or chemotherapy can be continued, with bilateral NSS performed in a two-step surgical procedure. This algorithm should be interpreted as a flexible decision-making aid requiring adaptation to tumor characteristics, treatment response, and multidisciplinary team assessment (Fig. 2).
